# Evaluation of a Health Information Exchange System for Geriatric Health Care in Rural Areas: Development and Technical Acceptance Study

**DOI:** 10.2196/34568

**Published:** 2022-09-15

**Authors:** Nils Pfeuffer, Angelika Beyer, Peter Penndorf, Maren Leiz, Franziska Radicke, Wolfgang Hoffmann, Neeltje van den Berg

**Affiliations:** 1 Section Epidemiology of Health Care and Community Health Institute for Community Medicine University Medicine Greifswald Greifswald Germany

**Keywords:** electronic health records, health information exchange, geriatrics, community-based participatory research, technical acceptance, usability, health information network, postacute care, patient-centered care

## Abstract

**Background:**

Patients of geriatrics are often treated by several health care providers at the same time. The spatial, informational, and organizational separation of these health care providers can hinder the effective treatment of these patients.

**Objective:**

This study aimed to develop a regional health information exchange (HIE) system to improve HIE in geriatric treatment. This study also evaluated the usability of the regional HIE system and sought to identify barriers to and facilitators of its implementation.

**Methods:**

The development of the regional HIE system followed the community-based participatory research approach. The primary outcomes were the usability of the regional HIE system, expected implementation barriers and facilitators, and the quality of the developmental process. Data were collected and analyzed using a mixed methods approach.

**Results:**

A total of 3 focus regions were identified, 22 geriatric health care providers participated in the development of the regional HIE system, and 11 workshops were conducted between October 2019 and September 2020. In total, 12 participants responded to a questionnaire. The main results were that the regional HIE system should support the exchange of assessments, diagnoses, medication, assistive device supply, and social information. The regional HIE system was expected to be able to improve the quality and continuity of care. In total, 5 adoption facilitators were identified. The main points were adaptability of the regional HIE system to local needs, availability to different patient groups and treatment documents, web-based design, trust among the users, and computer literacy. A total of 13 barriers to adoption were identified. The main expected barriers to implementation were lack of resources, interoperability issues, computer illiteracy, lack of trust, privacy concerns, and ease-of-use issues.

**Conclusions:**

Participating health care professionals shared similar motivations for developing the regional HIE system, including improved quality of care, reduction of unnecessary examinations, and more effective health care provision. An overly complicated registration process for health care professionals and the patients’ free choice of their health care providers hinder the effectiveness of the regional HIE system, resulting in incomplete patient health information. However, the web-based design of the system bridges interoperability problems that exist owing to the different technical and organizational structures of the health care facilities involved. The regional HIE system is better accepted by health care professionals who are already engaged in an interdisciplinary, geriatric-focused network. This might indicate that pre-existing cross-organizational structures and processes are prerequisites for using HIE systems. The participatory design supports the development of technologies that are adaptable to regional needs. Health care providers are interested in participating in the development of an HIE system, but they often lack the required time, knowledge, and resources.

## Introduction

### Background

Advanced age is associated with a higher morbidity risk and a higher risk for multiple comorbidities. Older patients are more likely to be affected by functional limitations and lose their independence and autonomy [1]. Morbidity, functional limitations, and symptoms in patients of geriatrics can vary widely. Thus, these patients are often treated by several health care providers with different tasks and competencies [2,3].

In Germany, the geriatric services of different health care professions, levels of health care provision (general and specialized care), and inpatient and outpatient health care are distinctively separated from each other with respect to planning, service implementation, access, and reimbursement. Specialized geriatric health care is provided by a variety of professions and includes inpatient and outpatient services [4,5].

As a consequence, there are significant communication and co-operation requirements associated with the provision of geriatric care. Especially in rural federal states such as the study region, Mecklenburg-Western Pomerania, specialized geriatric health care is rare and the distances are large, limiting close co-operation between health care providers and, hence, a comprehensive case management of the patients [6].

In rural areas, there is usually less access to health care for older people, and it is of lower quality compared with that available to urban patients even when considering the inconsistent definition of *rural* and possible interferences with other sociodemographic aspects [7,8]. As a result of demographic change, predominantly rural communities are often both declining in size and aging faster [9]. At the same time, the work environment in rural areas is often not very attractive to health care providers (eg, because of economic issues or working conditions). In addition, long distances are a major barrier to access to geriatric health care for older adults with multimorbidity and reduced mobility [7,9-11].

An analysis of problems and preferred solutions based on a questionnaire for German health care providers showed that the organizational and spatial separation of cotreating providers is one of the most urgent problems in rural areas. The respondents mostly preferred cross-professional networking to meet this challenge [12]. A study in the United States on older patients with comorbidities who needed a surgical procedure showed that information exchange between primary care providers and surgical providers is often discordant during transition, particularly the communication of the functional and social status of the patients [13]. Facilitating cross-institutional communication is a promising way to improve the quality and efficiency of geriatric health care. Information and communication technologies (ICTs) in health care such as electronic health records (EHRs) or health information exchange (HIE) systems jointly managed by all health care providers who are involved in the treatment of patients of geriatrics can be an option to support regional geriatric health care [3,14].

EHRs have a broad range of technical approaches and functionalities. The International Organization for Standardization defines the term EHR as a “repository of information regarding the health status of a subject of care, in computer processable form” [15]. For the International Organization for Standardization, facilitating continuous, efficient, and quality integrated health care is the primary goal of an EHR [15]. In addition to EHRs, an important key area is HIE, which allows health care providers to share and access clinical patient health information electronically across settings. HIE approaches have a number of benefits for health care, especially for patients with chronic illnesses, such as safer care, a reduction in the patients’ length of stay, fewer laboratory and imaging orders [16], and reduced mortality and serious adverse event incidence [17]. However, the resistance of health care providers [18,19], the difficulty of implementation in existing workflows [20], or a lack of interfaces with other digital patient documentation and information systems [21,22] can prevent the sustainable implementation of ICTs in practice.

**Figure 1:**
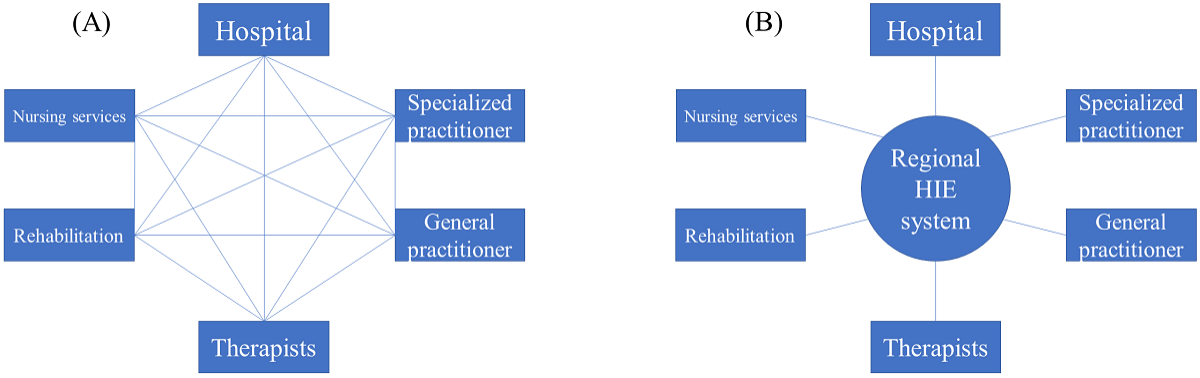
(A) The status quo: unilateral exchange of health information between practitioners who are typically involved in geriatric care in the status quo (eg, by mail or telephone). (B) Multilateral exchange via the regional health information exchange system. The links between the practitioners represent potential communication processes to share patient health information.

Figure 1 shows how the communication processes between geriatric health care providers can be streamlined through the use of a regional HIE system. The effectiveness of an HIE system, measured by the reduction in the quantity of potential communication and data transfers, is expected to increase with the number of providers engaged in the care of a patient.

### Research Questions

**Figure 2:**
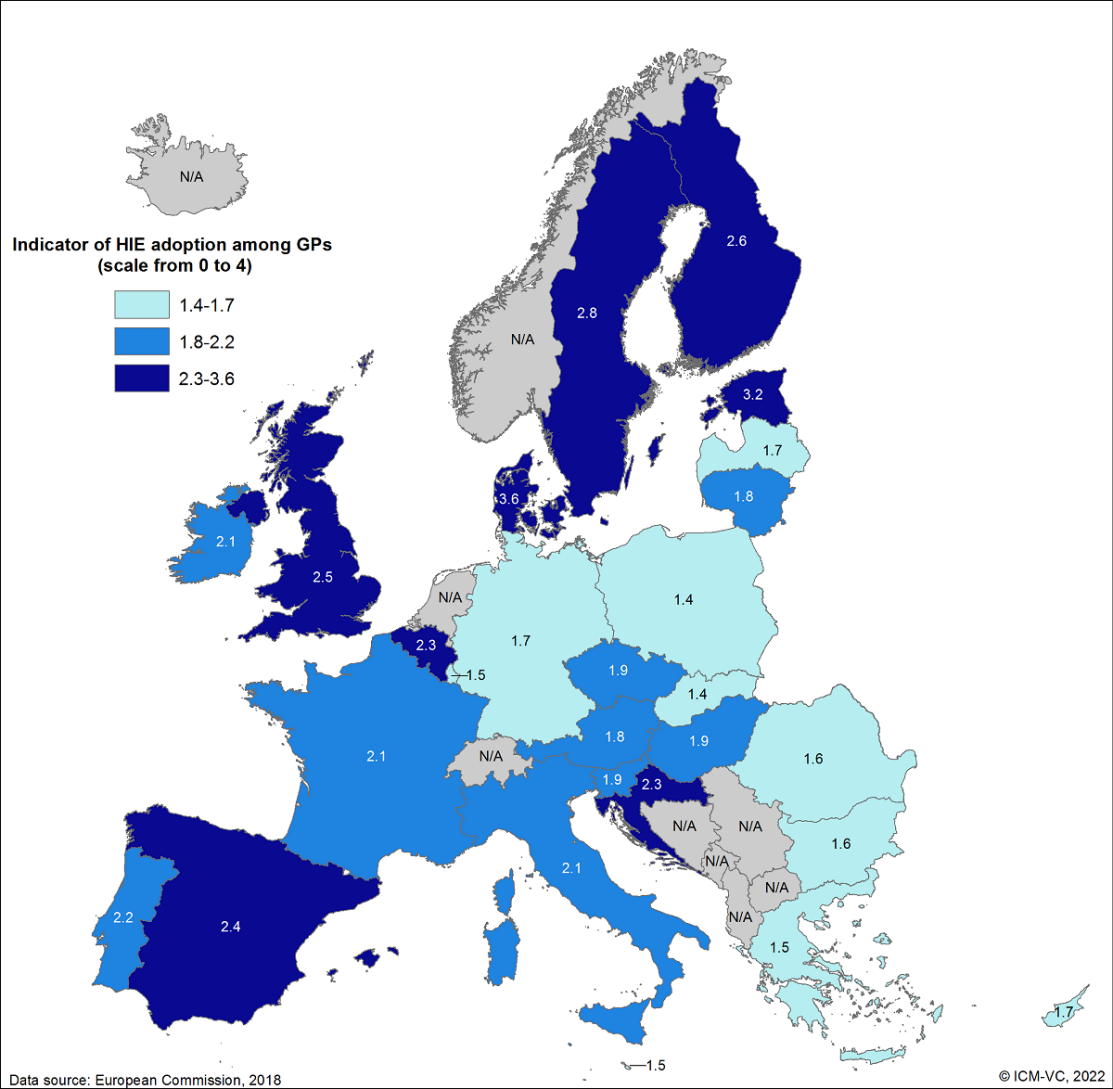
Health information exchange (HIE) adoption by general practitioners (GPs) in the European Union (EU) [23]. The scores reflects the share of GPs (n=5,793) who indicated the following state of HIE use in their practice: 0=not aware; 1=do not have it; 2=have it but do not use it; 3=use it occasionally; 4=use it routinely. ICM-VC: Institute for Community Medicine, Section Epidemiology of Health Care and Community Health; N/A: not applicable, because not a member state of the EU or no data available (eg, the Netherlands).

Recently, Germany passed several laws to foster the use of ICTs, such as the eHealth *Gesetz* (Act for Secure Digital Communication) in 2016 or the *Krankenhauszukunftsgesetz* (Act for a Future Program on Hospitals) in 2020. Particularly noteworthy is the *Patientendaten-Schutz-Gesetz* (Act for Protecting Electronic Patient Data) from 2020, which obliged statutory health insurance companies in Germany to provide EHRs for their members by 2021 at the latest. Health care providers are able to save patient health information, prescriptions, medical reports, and results in those EHRs, which can be accessed by patients via an app. However, Germany is lagging behind other European countries regarding the use and dissemination of ICTs (eg, in terms of the adoption of HIE systems by general practitioners [GPs]; Figure 2) [23] or the use of health IT (HIT) applications in hospitals [24]. Although approximately 89% of German GPs’ practices are connected to the telematics infrastructure, which enables HIE and the use of other HIT applications [25], the communication between GPs and hospitals is still mainly paper-based [24].

A study on an HIE system in combination with automated clinical event notifications supporting multidisciplinary care coordination for patients of geriatrics has shown that the system can reduce potentially avoidable admissions and duplicate testing [26]. However, in the United States, between 2012 and 2014, a decline in planning and operating efforts was observed in the field of HIE systems [27].

A study on users’ acceptance of an HIE system to coordinate the care of patients with chronic illnesses and mental comorbidities has shown that contextual factors, such as various motivational factors, the level of trust between patients and physicians, or incomplete transmission of information, may reduce the willingness of individuals to use HIE systems [28]. However, only a few studies have examined the efficiency and effectiveness of HIE systems [29].

Therefore, the aim of this study was to develop and evaluate a regional HIE system that supports information exchange in rural geriatric care. To counter the aforementioned barriers to implementation, the development followed a community-based participatory research (CBPR) approach that sought to identify and incorporate specific needs as well as the practical knowledge of the affected geriatric health care providers.

Considering the development of the regional HIE system, the research questions were as follows: (1) How can the quality of co-operation between health care providers and between health care providers and researchers be described? (2) What motivates the participating health care providers to engage in the development of the regional HIE system? (3) What barriers and facilitators can be identified with regard to the use of the regional HIE system? (4) What practical feasibility issues can be observed? (5) What use cases can be supported by the regional HIE system? (6) What functions should the regional HIE system provide to support regional geriatric health care involving all relevant regional health care providers? (7) How is the users’ acceptance of the regional HIE system and what factors may affect it?

## Methods

### Overview

Following the CBPR approach, this study used a flexible, iterative, and open-ended mixed methods approach [30] to integrate the knowledge and practical insights of the participating health care providers into the development of the regional HIE system [31,32]. The following stages were conducted: identification of suitable geographic regions for implementing the regional HIE system, identification of regional stakeholders, compilation of specific regional problems of geriatric health care, development of a common workflow, definition of the specific needs of the stakeholders, development of the regional HIE system, and usability testing. Multimedia Appendix 1 depicts the development and research activities in detail. Qualitative methods were applied during the entire course of development. At the end of the project, a survey was conducted.

### Ethics Approval

The ethics board of the University Medicine Greifswald reviewed and approved this study (BB 083/18).

### Qualitative Phase

#### Participants and Recruitment

The first step was the identification of suitable regions within Mecklenburg-Western Pomerania. Preferred regions were those with geriatric facilities that were already co-operating in a network of health care providers. Health care providers in each region were recruited based on an open-ended, casual sampling strategy, including snowball sampling, as this allows for a sampling of natural interactional units [33]. At the beginning, health care providers identified as central to regional geriatric health care were invited to jointly develop an EHR. This initial group was then asked to bring in further interested co-operation partners from different health care professions and sectors.

To organize and conduct the meetings between researchers and participating health care providers, CBPR principles according to Israel et al [34,35] were followed. These principles aim to reconcile the interests of the researchers with those of the users, such as building on strengths and resources within the community, recognizing the participating networks as units of identity, sharing decision-making, jointly disseminating the results, and presenting the regional HIE system to other interested health care providers.

For the usability tests of the regional HIE system, patients of the participating geriatric health care providers were included after they provided informed consent. Following the definition of patients of geriatrics of the German expert associations for geriatric care, eligible persons were patients aged >70 years and who had at least two geriatric-typical syndromes or who were aged >80 years [36]. Geriatric-typical characteristics include, for example, frailty, decubitus, and tendency to fall [36,37].

#### Setting

The study took place between January 2018 and October 2020 in the northeast of Germany (federal state of Mecklenburg-Western Pomerania). The setting included inpatient as well as outpatient geriatric care. In Mecklenburg-Western Pomerania, geriatric rehabilitation clinics and acute stationary hospitals are allowed to provide inpatient geriatric care. Outpatient geriatric care can be provided by GPs with or without special training in geriatric care working together with therapists’ practices.

#### Data Collection and Analysis

During the initial workshops in each focus region, the participants were asked to identify the relevant functions of HIE in geriatric care (eg, electronic case report forms [eCRFs] on diagnoses, medication history, or certain assessment instruments). The results of the workshops were used to design the regional HIE system based on a pre-existing system, the so-called eHealth platform of the University Medicine Greifswald.

Before the participants started testing the regional HIE system, they received training at the workplace on how to use its basic functions. Each partner received a personal client certificate and an individual user account. The participants were asked to test the functions and notify the researchers regarding which adjustments should be made and which additional functions they would need for use in practice. This process was repeated iteratively several times until the regional HIE system provided a comprehensive set of functions that met the needs of geriatric care. User acceptance and usability aspects were simultaneously assessed using the regional HIE system for the HIE of representative (ie, geriatric) cases of the participating health care facilities for test purposes. Usability issues were identified based on the feedback of the users after these tests.

Qualitative data were collected by means of participant observation and informal interviews during the workshops and other meetings to characterize the co-operation within each focus region; identify barriers to and facilitators of HIE in geriatric care; and evaluate the participants’ acceptance of the regional HIE system, which included usability aspects. Moreover, qualitative data on the participants’ motivation to engage with the regional HIE system were gathered using free-text items in a questionnaire. Especially for obtaining insights into workflow and usability issues of HIE systems, qualitative methods such as observations and interviews were seen as useful [38]. An approach using observations in combination with informal interviews is relatively unobtrusive and, therefore, was easy to integrate into workshops and meetings with practice partners. It also had the advantage of preventing participants from perceiving themselves as study objects, thereby offering the opportunity to observe actions or opinions under everyday conditions. Observation is a promising method to evaluate complex objects of investigation such as interactions within a group of different people over a certain period as other methods would not or would only indirectly provide answers to the research questions [38].

Owing to the coincidental nature of observations and informal interviews [39], no interview transcripts exist. Observations and interview notes were taken by the researchers right after the contacts in a project diary for each focus region, with information about the time, participants, and content of the contacts. To report the qualitative data in our research, we adhered to the SRQR (Standards for Reporting Qualitative Research) [40].

Following the guidelines of the SRQR, the qualitative findings and results of the standardized questionnaire were cross-checked to ensure the trustworthiness of the qualitative data. To increase reliability, the following means were used: if more than one researcher attended a project meeting with the participating health care providers, the observations were discussed afterward. After the data collection phase, the project diaries were checked for incoherencies by two other researchers (AB and PP) involved in the project. Furthermore, all email correspondence and phone contacts with the participants were documented, which served as an audit trail for the research activities.

Project diary entries were categorized using inductive content analysis. The data were analyzed using MAXQDA (version 10; VERBI Software Consult).

### Quantitative Phase

#### Sampling

Convenience sampling was used to select the survey participants. As the study was interested in the participants’ acceptance of the regional HIE system, participants had to attend at least one regional HIE workshop or meeting with the research team. Furthermore, the participants had to be involved in geriatric care. However, there were no restrictions with respect to their profession (medical, therapeutic, and nursing staff) or sector of the health care system (eg, practices or hospital).

#### Setting

The survey was conducted in health care facilities that are usually involved in geriatric care and that participated in the development of the regional HIE system. Inpatient as well as outpatient facilities were included.

#### Data Collection and Analysis

**Figure 3:**
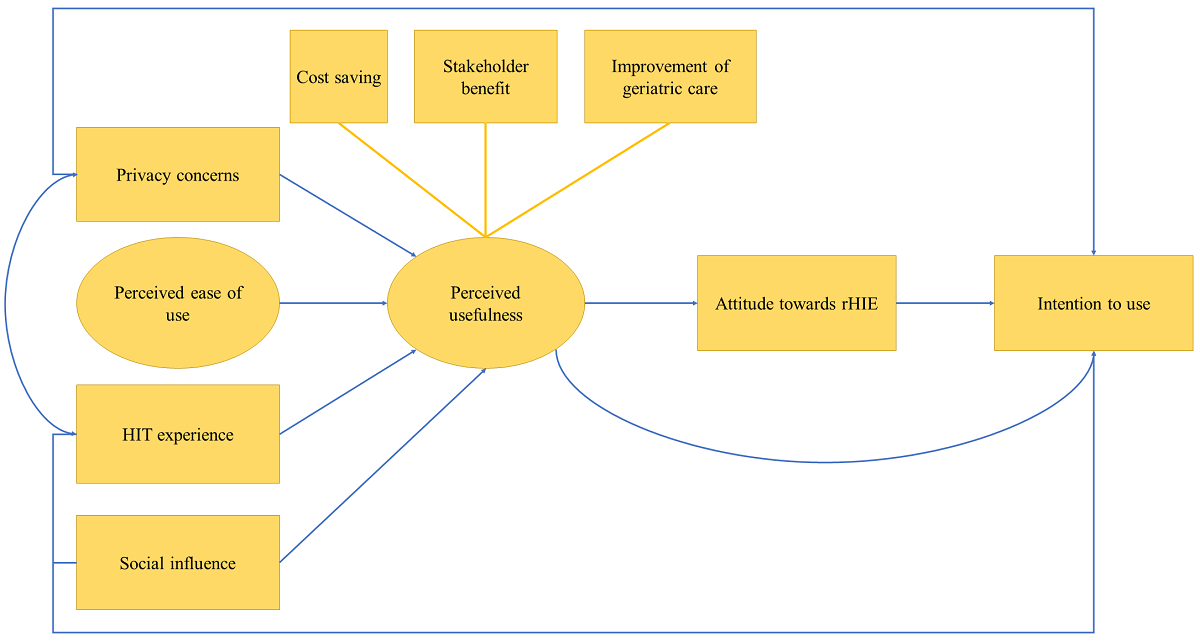
Adapted and tested technology acceptance model for health ITs (HITs), own illustration, based on Steininger and Stiglbauer [41]. rHIE: regional health information exchange.

The questionnaire sought to evaluate the satisfaction of the participants with the developmental process, their motivation for participating, their attitude toward the regional HIE system, and the factors affecting their intention to use it in their working practice. To evaluate the participants’ acceptance of the regional HIE system, items from an adjusted technology acceptance model (TAM) [41] were used. This is an adapted model specifically describing influential factors for the acceptance of a shareable EHR, which focuses on the intention to use rather than on actual use. Thus, it is a suitable model for considering technologies that are still in the preprototype stage. This model is shown in Figure 3.

The survey, as a quantitative method, was seen as a suitable means to objectively determine the aforementioned variables and cross-check the results of the qualitative survey.

The questionnaire included 35 questions regarding the status quo of communication in geriatric care (eg, the current quality of communication, perceived communication costs, frequent communication partners, frequently missing patient health information, current communication means, and local electronic medical record [EMR] systems in use). This was followed by a set of statements on the acceptance and perceived usability of the regional HIE system according to the TAM (Figure 3) and the assessment of the CBPR co-operation. The statements were to be evaluated using a 5-point Likert scale (“strongly agree”-“strongly disagree”). The last section consisted of questions asked to obtain demographic details about the participants (eg, occupation, affiliation to a health care facility, membership status in medical networks, age, and sex). The questionnaire was pretested by 5 research colleagues. A descriptive analysis of the quantitative data was conducted, and the results were presented both in total numbers and in relative percentages. Free-text answers were categorized using inductive content analysis.

### Technical Infrastructure

The so-called eHealth platform of the University Medicine Greifswald served as the technical basis for the development of the regional HIE system. The eHealth platform includes a user interface (c37.CaseBoard by celsius37.com AG) and a database back end consisting of an Orchestra server (Orchestra eHealth Suite; version 18.2.1; x-tention) supporting Integrating the Healthcare Enterprise standards, such as Cross-Enterprise Document Sharing, which allows for cross-organizational exchange of medical documents and information; Patient Identifier Cross-Referencing for cross-organizational patient identification; Cross-Enterprise User Assertion for cross-organizational user authorization; and Audit Trail and Node Authentication, which allows for an audit trail and node authentication.

X-tention Orchestra structures and merges data, including record linkage, in the main database, whereas c37.CaseBoard, as the user interface, enables health care professionals to edit and manage patient health and treatment information. The original intention of the project was to use the eHealth platform for exchanging patient health information between subsidiary facilities affiliated with the university hospital (eg, radiological images taken by an affiliated walk-in clinic).

## Results

### Qualitative Results

#### Characterization of Participants and Focus Regions

**Figure 4:**
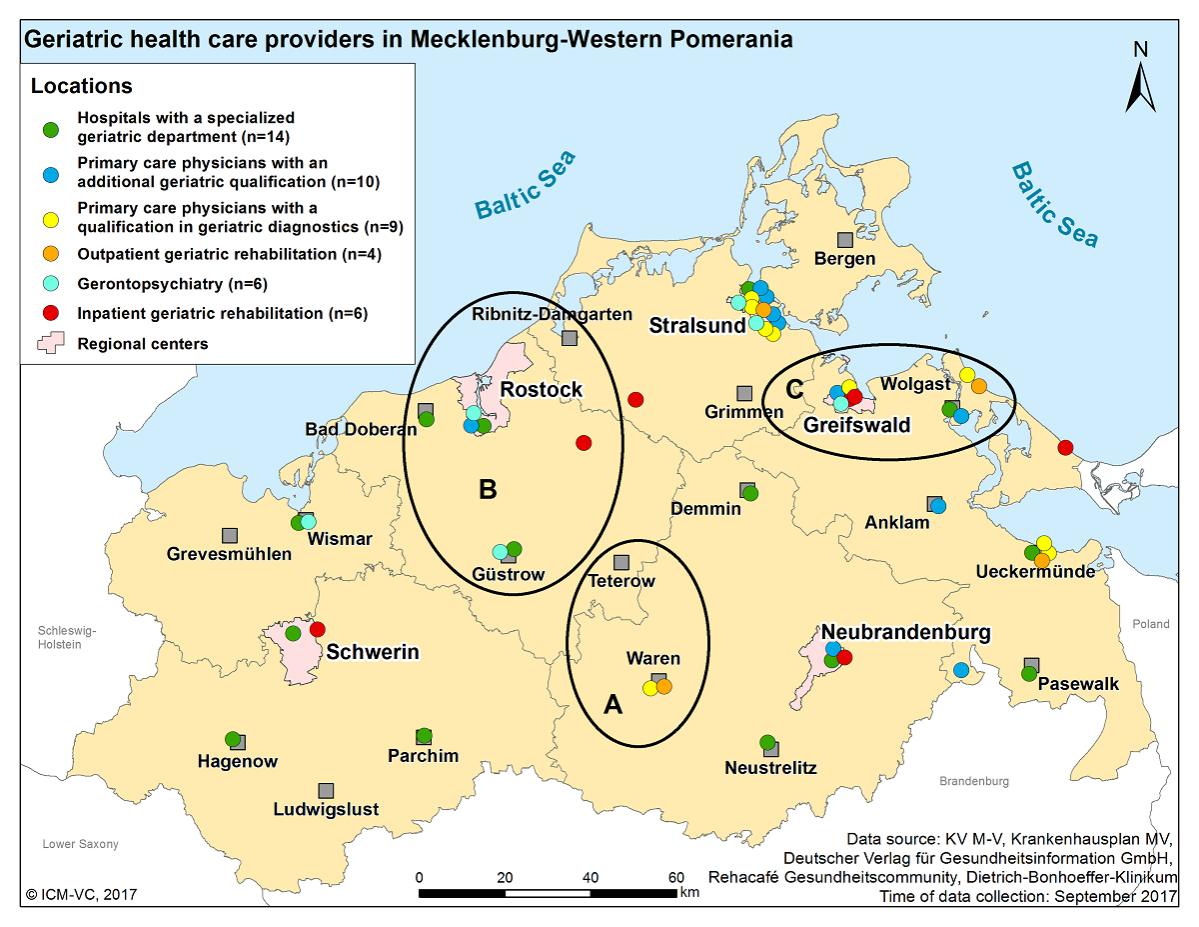
The three focus regions (A, B, and C) involved in the development of the regional health information exchange system. ICM-VC: Institute for Community Medicine, Section Epidemiology of Health Care and Community Health ; KV M-V: Association of Statutory Health Insurance Physicians in Mecklenburg-Western Pomerania; MV: Mecklenburg-Western Pomerania.

Health care providers from 3 focus regions participated in the development and implementation of the regional HIE system (Figure 4). In region A, local GPs, a specialized GP (a primary care physician with a qualification in geriatric diagnostics or an additional qualification in geriatric care), and an acute inpatient hospital without a specialized geriatric department were involved. In region B, GPs, a specialized geriatric GP, a hospital with a specialized geriatric department, and an inpatient geriatric rehabilitation clinic participated. In region C, a hospital with a specialized geriatric department collaborated with a geriatric day clinic and local GPs.

In total, 22 people from all 3 focus regions participated in CBPR activities on developing the regional HIE system. All 22 were included in the participant observations and informal interviews. Multimedia Appendix 2 characterizes the 3 participating focus regions in detail and shows that different health care professions and facilities participated in the workshops and meetings on the regional HIE system during the developmental process. Each region contributed geriatric qualifications and specializations to a different extent. The extent of networking within each region also varied. The networks in regions A and B had a formal co-operation agreement (which also included joint activities beyond the scope of mere patient care), whereas the network in region C was of an informal nature and, thus, solely restricted to the joint care of patients that is typical in the health care system (eg, because of the transfer of patients between different sectors or health care facilities). One of the formal networks had a focus on geriatric care (region B), and the other had a focus on general care with mainly GPs as members. The network from region B was the only network with a network coordination office, which organizes multi-professional task forces on certain issues of cross-organizational health care in the region.

A total of 12 workshops were conducted between January 2018 and October 2020. Multimedia Appendix 3 depicts how many workshops were conducted in each region and what achievements could be made.

To test usability, 50 patients were recruited between June 2019 and October 2020 in region B. In regions A and C, practitioners used test data sets for usability testing of the regional HIE system.

#### Relationship Between Participants and Between Participants and Researchers

On the basis of observational data, Multimedia Appendix 4 characterizes the relationship between the participating health care providers in the 3 focus regions and the relationship between the participants and the researchers considering the CBPR principles. It was found that co-operation with the network in region B was the best with regard to continuity, trustworthiness, and the strategic orientation of the collaboration.

#### Health Care Providers’ Motives for Participation

Table 1 shows the CBPR partners’ most important reasons for participating in the regional HIE project. The improvement of the quality of care, promotion of cross-sectoral co-operation, and reduction of administrative costs for patient documentation were the strongest motives for participating. Quality of care refers to patient-related outcomes, including rehospitalizations, adverse drug effects, or need for nursing services. Promoting cross-sectoral co-operation means the general improvement of communication and information exchange between different health care facilities treating the same patients. Lower communication and documentation costs refers to the expectation of the participating facilities that they will be able to reduce their administrative costs associated with sharing or documenting patient health information.

**Table 1:** The project partners’ motives for participation in the regional health information exchange project. Respondents’ free-text answers from the questionnaire (categorized; N=11).

Motive	Partners, n (%)
Quality of care	6 (55)
Promoting cross-sectoral co-operation	6 (55)
Lower communication and documentation costs	5 (45)
More efficient use of resources in health care	3 (27)
Better availability of information	2 (18)
Proxy co-operation (eg, improvement of business relations)	1 (9)
Uniform cross-divisional discharge management	1 (9)
Patient-centered focus on overall health	1 (9)
Other	1 (9)

#### Identified Use Cases and the Extension of the eHealth Platform

A total of 3 use cases of the regional HIE system were identified (Textbox 1).

The following functions were identified and implemented into the regional HIE system: assessment eCRFs (specific geriatric assessment instruments such as the Barthel Index, Mini-Mental State Examination, and Mini Nutritional Assessment), diagnosis eCRFs (International Classification of Diseases, 10th Revision, and German codes for principal and secondary diagnoses), medication eCRFs (prescribed substance, dose, dosing time, and purpose), an eCRF for contact persons (contact information of the responsible nursing service, GP, caregiver, and relatives), assistive device eCRFs (assistive devices already used by the patient and those newly prescribed), and an eCRF on powers of attorney and the living will of the patient. In addition, users can share any medical documents (eg, medication plans, physician’s letter, and discharge letters) by scanning and uploading them as PDFs to the regional HIE system.

**Use cases of the regional health information exchange (HIE) system.**

**Use cases**

- Discharge management: the regional HIE system should provide treatment information (eg, diagnoses and results) of an inpatient stay for other involved health care professionals as soon as this information has been collected. Hence, general practitioners (GPs) will be able to coordinate the subsequent treatment of their patients more effectively and at an early stage of care.
- Outpatient geriatric treatment: GPs or practitioners specialized in geriatrics should be able to share information on assessment results, prescribed medications, assistive devices, therapies, care needs, and social medical information.
- Emergency care: previously recorded patient health information would be accessible in an emergency independent of time and location.

After the extension of the former eHealth platform, authorized geriatric health care providers are able to access the regional HIE system for exchanging health information of their patients with other health care providers involved in the treatment but not with the patients themselves.

**Figure 5:**
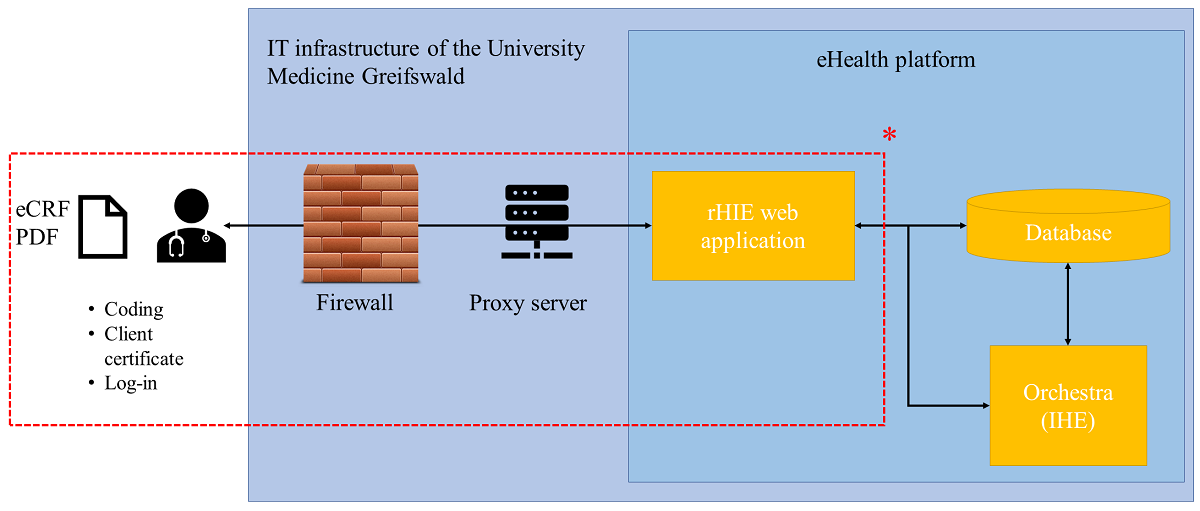
Technical infrastructure of the eHealth platform. Authorized users can exchange patient health information via electronic case report forms (eCRFs) or as digitized documents (eg, PDFs). IHE: Integrating the Healthcare Enterprise; rHIE: regional health information exchange. *New components of the rHIE system added to the pre-existing eHealth platform.

In this project, digitized documents were only exchanged as scanned PDFs, but a structured data exchange based on Fast Healthcare Interoperability Resources standards is theoretically possible if the local EMRs of the participating health care facilities support Fast Healthcare Interoperability Resources and have the required interfaces. All the involved practitioners can communicate directly by using a comment function. Figure 5 depicts how the technical infrastructure of the eHealth platform has been extended.

Screenshots of the functions of the regional HIE system are provided in Multimedia Appendix 5.

#### Barriers to and Facilitators of the Use of the Regional HIE System

On the basis of the observational data, the following barriers to and facilitators of the use of the regional HIE system were identified.

A total of 13 barriers were identified (Multimedia Appendix 6). Major barriers were as follows: some users considered the regional HIE system to be a comprehensive EHR rather than a pure HIE platform; therefore, they found it too laborious to use the regional HIE system in parallel with their local and mainly paper-based patient records. A participant criticized that the parallel structures of local patient records and the regional HIE system meant that the availability of information for other collaborating facilities still depended on when health care providers actually transferred data to the regional HIE system. Thus, whether the patient health information on the regional HIE system was available in time still depended on local workflows. In addition, 9% (2/22) of the participants proposed improving the regional HIE system by adding a notification function that informs other health professionals involved in the treatment if patient information is updated or new documents are uploaded.

Moreover, users complained about ease-of-use aspects and technical issues that negatively affected the use of the regional HIE system, such as bad internet connectivity, unnecessary mandatory fields in the eCRFs, problems with browser settings, or the overly complex registration process.

The fact that there is not yet region-wide use of the regional HIE system and a lack of trust among users were seen as further barriers to the implementation of the regional HIE system. Owing to the patients’ right to choose their practitioner freely on the one hand and the complex user registration process on the other, it was not always possible to share patient health information with all health care professionals actually involved in the treatment. Finally, 14% (3/22) of the participants did not want to use the regional HIE system as they feared too much transparency in terms of their working procedures and outcomes.

In total, 5 facilitators were identified (Multimedia Appendix 7). One of the main facilitators was the adaptability of the regional HIE system to local needs by using a modular structure and customizable eCRFs. In contrast to EHRs of different statutory health insurance companies, which have been developed recently, the regional HIE system is open for all patients regardless of their membership of certain insurance companies, which makes adoption of the regional HIE system to support local information exchange easier. Moreover, the web-based design enables facilities to exchange information via the regional HIE system regardless of their individual technical infrastructure. Furthermore, participants who saw the regional HIE system as an HIE system for transferring only certain information relevant to treatment rather than a comprehensive EHR of patient health information were more open-minded about the use of the regional HIE system in practice. Trust among the participants within their local health care networks also increased the use of the regional HIE system. Finally, high computer literacy of the users was seen as helpful in the implementation of the regional HIE system.

### Quantitative Results

#### Survey Participants

All 22 participants in the CBPR workshops were asked to complete a questionnaire at the end of the project. A total of 12 questionnaires were filled out (12/22, 55% response rate). There were several reasons for nonresponse. A total of 14% (3/22) of the participants filled out the questionnaire together with another colleague from their team and did not send back their own questionnaires. In 18% (4/22) of the cases (all from region A), the head of the network insisted on performing the testing as a representative of the other participating GPs. Therefore, the other GPs did not feel responsible or were not able to answer the questions asked in the questionnaire. In 14% (3/22) of the cases, the reasons for not answering were unknown.

In region A, 100% (3/3) of the participants were aged between 51 and 60 years. In region B, 60% (3/5) of the participants were aged between 41 and 50 years, and 20% (1/5) were aged between 21 and 30 years. In region C, 25% (1/4) of the participants were aged between 41 and 50 years, 25% (1/4) were aged between 31 and 40 years, and 25% (1/4) were aged >60 years. A total of 17% (2/12) of the respondents (1/2, 50% from region B and 1/2, 50% from region C) did not provide their age. Thus, participants from region A were, on average, older than the other participants. Region B was the region with the youngest participants on average.

#### Status Quo of HIE Processes in Regional Geriatric Health Care

Of the 12 respondents, 8 (67%) often or always depended on information from other health service providers in the treatment of their patients, and 1 (8%) did not respond. The respondents indicated that the following health care facilities were central for the cross-sectoral HIE in geriatrics (multiple choices were possible): hospitals (9/12, 75%), nursing care services (9/12, 75%), GPs (8/12, 67%), speech and occupational therapists (7/12, 58%), and geriatric rehabilitation and day clinics (4/12, 33%).

Information and patient data that were often not (immediately) available but needed for further treatment of patients of geriatrics were as follows (multiple choices were possible): information about prescribed assistive devices (5/12, 42%), vaccination (4/12, 33%), social situation (4/12, 33%), self-medication of the patient (4/12, 33%), geriatric assessments, and discharge letters (3/12, 25%).

Furthermore, the questionnaire respondents were asked to assess the quality of the current HIE. Of the 12 participants, 5 (42%) rated the quality of information exchange as good, 5 (42%) rated it as neutral, 1 (8%) rated it as bad, and 1 (8%) did not answer.

Of the 12 participants, 8 (67%) considered the effort and expenses involved in the current cross-institutional HIE as high to very high, and 3 (25%) felt that the demands were reasonable.

A total of 67% (8/12) of the participants stated that they were using paper-based records supported by electronic data processing. A total of 33% (4/12) of the participants (1/4, 25% from region A and 3/4, 75% from region B) answered that they were using comprehensive digital records. Of those 4 participants, 1 (25%) from region B was using a digital record with the ability to share patient data with other facilities.

Considering the means currently used for exchanging patient health information, 12 participants, with 1 missing, responded as follows (multiple responses were allowed): 10 (83%) were using mail or fax, 9 (75%) were using phones, 5 (42%) were using email, and 1 (8%) was using WhatsApp or similar apps. No participant used SMS text messaging or HIE systems. In all regions, half of the participants (2/3, 67% from region A; 3/4, 75% from region B; and 1/5, 20% from region C) were using only conventional, nondigital means of communication (mail, fax, or phone) for exchanging medical information.

#### Acceptance of the Regional HIE System

Multimedia Appendix 8 shows different aspects of the TAM 2 and to which extent the participants agreed that the regional HIE system could fulfill these aspects in practice. The participants were mostly skeptical regarding technical (3/12, 25%) and organizational integrability (3/12, 25%) and being able to provide the necessary human resources needed to use the regional HIE system in practice (5/12, 42%).

The participants mostly agreed that they were appropriately informed of the advantages and disadvantages of the regional HIE system (7/12, 58%). Most believed that the regional HIE system was able to improve different aspects of the provision of geriatric health care (quality of care: 8/12, 67%; availability, completeness, and timeliness of important medical information: 7/12, 58%; and continuity of care: 5/12, 42%). The regional HIE system was also considered by 42% (5/12) of the participants to reduce the expenses incurred for documentation and communication in comparison with the status quo of HIE.

Nearly half of the participants agreed that the regional HIE system guaranteed an appropriate level of privacy protection for both patient (5/12, 42%) and provider (5/12, 42%) data. The same proportion of participants agreed that colleagues from their own and other facilities found the regional HIE system useful (5/12, 42%). Half of the participants planned to continue using the regional HIE system after the project ended (6/12, 50%). Nearly half also agreed that they would recommend the use of the regional HIE system to other colleagues (5/12, 42%).

The participants from region A were the most critical about using the regional HIE system in practice after the end of the project. A total of 67% (2/3) of the participants from region A indicated that they would not use the regional HIE system in practice. However, in region B, 60% (3/5) of the participants and, in region C, 50% (2/4) of the participants agreed that they would use the regional HIE system in practice. The other participants from regions B and C gave a neutral response.

Although the regional HIE system was developed by involving the regional geriatric experts, after finishing development, only 2 of them agreed that the regional HIE system had appropriate functions to support HIE in geriatric care.

## Discussion

### Principal Findings

Regarding the use of the regional HIE system in practice, the following barriers were identified: lack of trust owing to the implicit disclosure of own treatment methods to other users, missing regional geriatric network structures, time constraints, limited human resources, differences in computer literacy, and some ease-of-use issues. An overly complicated registration process for health care professionals and the patients’ free choice of the treating health care provider can, in turn, result in an incomplete exchange of patient health information via the regional HIE system. Among the participants, acceptance of the regional HIE system was high but varied between the 3 focus regions. The status quo of pre-existing HIE systems was at a low level in all regions.

According to the observational data, a concern was that the use of the regional HIE system could result in an increased workload for the participants instead of reducing the effort required for documentation as there is no automatic data transfer between the regional HIE system and the local EMR yet. Chronaki et al [42] also found that, because of individual resistance to innovations, changes in workflow related to the implementation of an EHR could lead to a heavier workload for health care professionals at the beginning. Switching from one system to another, insufficient financial resources, and the absence of computer skills were also identified as barriers in a study on factors that affected the uptake of an EHR by GPs in Ireland [43]. In addition to a lack of technical support, a lack of support at the management, colleague, or even political level can be a barrier to the implementation of an EHR [44,45].

The exchange of information between practice and hospital information systems and a regional HIE system is generally a challenge in Germany as there are >110 different practice management systems and also a variety of hospital information systems. In addition, most respondents (10/12, 83%) still mainly used paper-based records, albeit in combination with electronic data processing such as practice management software products. As a consequence, full integration of the regional HIE system into local EMRs is challenging. However, the amount of time saved by using an EHR increases with the level of interoperability of the EHR system [46], so further development of the regional HIE system should focus more intensively on interoperability and building up interfaces with other systems.

Some participants (2/22, 9%) were unsatisfied with the development of the regional HIE system as it took too much time. Moreover, they felt that they had too little influence on the development. However, the same participants had very high expectations (eg, automatic synchronization between the different local EMRs and the regional HIE system) and were generally skeptical of the project. It is known that staff skepticism, a lack of clinical leadership, a vendor whose products are not ready on time [47], and unfulfilled expectations are barriers to the implementation of EHRs [48]. Pagliari et al [49] outlined that clinicians’ mistrust of e-communications could also be a barrier.

Some participants (1/22, 5%) were concerned about patient and provider privacy. Rosen et al [50] suggested that physicians fear there might be a quality assessment based on their EHR use data, which might lead to a low uptake among physicians who, for instance, stick to more traditional referral processes. Hackl et al [51], based on interviews with Austrian physicians, concluded that there are serious concerns that EHR data could be used against the participating physicians. Ford et al [52] recognized a threat to physician autonomy and concluded that, despite an existing EHR system, this could result in a lack of information sharing. Therefore, a role- and rights-based access policy should be integrated into the regional HIE system, which allows the owners of the patients’ medical documents or records to release them to predefined subsets of health care providers, for example, exclusively to nursing services, only family physicians, or to a combination of these user groups. By contrast, the patient should be able to grant and control health care providers’ individual access to the regional HIE system (eg, using a personal identification number or managing access settings via the patient’s own account). This would also solve the problem that not all relevant health care providers can be added to the HIE system in advance.

The following facilitators of the use of the regional HIE system were identified: adaptability and modular structure of the regional HIE system, web-based design, use of the regional HIE system as an HIE system, trust among the users of an HIE system, and computer self-efficacy. Although the web-based design does not solve the interoperability problem of a scattered HIT landscape, it was identified as a facilitator of the use of the regional HIE system as it allows health care providers with different technical resources to exchange health information. It helps overcome the problems associated with the existence of various kinds of patient records and IT systems (eg, paper-based vs electronic records). Another study also highlighted that the federated web-based design is a facilitator as it presupposes less trust among the participating users because each user retains the control of their own data [53].

Another facilitator was that the regional HIE system, in contrast to EHRs offered by statutory health insurance companies, can be used by all patients regardless of their individual insurance company. The regional HIE system also allows for HIE between health care providers involved in the treatment without it being restricted to certain medical information and results or by whether the patient uses the EHR function provided by their health insurance company. In contrast to EHRs, clinical data transferred via HIE systems follow the patient electronically across delivery settings and, thus, HIE systems are more able to improve care coordination [14].

Moreover, the participatory design can be seen as a facilitator of the adoption of the regional HIE system as it enables health care providers of a certain region to adapt the HIE system to the specific local geriatric health care needs.

Comparing the individual focus regions, it is remarkable that the collaboration with participants from region B was the best in terms of continuity of co-operation, strategic planning, network identity, engagement in participatory activities, and other CBPR aspects. In contrast, collaboration on the development of the regional HIE system was the worst in region A and average in region C. Most of the participants from region B (3/5, 60%) were also convinced of the benefits of the regional HIE system for local geriatric care and indicated that they would use it in practice after finishing the project, whereas participants from region A (2/3, 67%) in particular were more skeptical and mainly indicated that they would not continue to use the regional HIE system. A reason seems to be that practitioners trained in geriatrics and geriatric-focused networks were more open-minded about participatory designs and more often saw the urgency of cross-organizational information exchange to improve geriatric care outcomes. Moreover, the presence of a network coordination office, a nonhierarchical organization, and the leadership of a geriatric-focused clinic in region B could have facilitated development and implementation in the region.

Participants engaged in a network that seeks to develop and improve regional interdisciplinary geriatric concepts seem to be more interested in the regional HIE project than those who are going at it alone. A reason for this could be that these participants are more sensitized to the communication problems in the current health care system. Another reason may be that they already have a clear concept of what is needed to improve cross-sectoral communication. Mostashari et al [54] showed that already existing team-based care strategies were a good prerequisite for a successful implementation of EHRs.

Participants’ individual characteristics, such as age or computer literacy, could have been important factors affecting the acceptance of the regional HIE system as participants in region B were, on average, the youngest and a participant from region B had high computer literacy, whereas a participant from region A had doubts about using HIT innovations in his practice. Another study showed a significant negative correlation between health care workers’ age and their perception of telemedicine’s significance [55]. Moreover, previous studies have identified computer anxiety as a barrier [56] and computer self-efficacy [57] as a facilitator of the adoption of EHRs [58].

The technical state of HIE seems to be at a low level in all regions, which would be consistent with the general relatively low level of HIE adoption in Germany compared with other European countries. The participants had mainly used nondigital means for exchanging medical information, such as mail, phone, or fax, or used less secure and unilateral means of communication that made it difficult to verify the authenticity of the content and the sender, such as email or WhatsApp. Another survey on outpatient care providers’ electronic exchange of health information identified partner readiness and clinicians’ previous familiarity with HIT systems as the most important predictors for HIE system use [59]. Thus, the fact that the intention to use the regional HIE system was at a lower level in regions A and C might be explained by the participants having less previous experience using HIT systems.

### Strengths and Limitations

Especially for gaining insights into issues related to workflow and the acceptance of the regional HIE system, observations in combination with informal interviews proved to be a relatively unobtrusive approach and could easily be integrated into workshops and meetings with the practice partners. The mixed methods approach was also suitable for the evaluation of complex objects of investigation such as interactions within a group of different people over a certain period.

Furthermore, the CBPR approach helped address technical as well as organizational issues of implementation that came up during the course of development. Moreover, the participative design helped create functions that were adequately adapted to the regional needs of the providers. The CBPR strategies also seemed to have a positive effect on the users’ acceptance.

With the support of the participating health care providers, the development of the regional HIE system was effective. Participants brought in their social capital, reputation, and knowledge. In addition, researchers and practitioners used mutual symposia and workshops to leverage the synergies between research and practice.

This study has some limitations. It was based on a small number of very heterogeneous health care providers. The results are not representative of the entire community of geriatric health care providers or the health care system in general. Variances in the EHR and HIE infrastructure between the included focus regions are not known. Although a survey collecting quantitative results was conducted, the small sample did not allow for any inferential analysis of the results. However, the results provide a good picture of the regionally different structures of health care provision and associated facilitators of and barriers to the implementation of a regional HIE system.

Even though the CBPR approach seeks to involve all affected stakeholders equally, this was not always possible during the workshops and meetings because of the time constraints of certain participating health care providers. However, the participants represented a comprehensive and inclusive sample of health care providers who were usually involved in geriatric treatment. Observation data were cross-checked with survey data. This enabled a comprehensive and in-depth understanding of communication processes in regional geriatric care, the EHR functions needed, and the users’ acceptance issues.

### Conclusions

In summary, the time and effort required to build the necessary trust for a CBPR approach can be seen as the greatest barrier to the participatory design of a regional HIE system. Participative processes and communication efforts (eg, feedback groups, workshops, and training of participants) and the recognition of nonscientific institutions as eligible co-operation partners are necessary for a successful project.

In regions where CBPR collaborations could be established, the development of the regional HIE system was successful as the use cases for it could be identified directly based on the needs of the regions, the functions of the regional HIE system could be adequately designed, and there was a higher degree of acceptance among the users than in other regions. Meta-analyses of a larger sample of studies aiming to develop and implement HIE could provide more evidence to determine whether CBPR approaches are generally more suitable for increasing users’ acceptance.

In the future, further stakeholders should be involved in the implementation and further development of the regional HIE system. In addition, more research is needed on questions such as how to adequately remunerate HIE use, which legal adjustments are needed, and how to facilitate cross-sectional co-operation in a fragmented health care system. Finally, the regional HIE system should be evaluated when used with a more general purpose such as a multi-setting environment for more generalizable results on its usability and acceptance.

## Appendix

Multimedia Appendix 1 Details and timeline of the developmental process. HIE: Health information exchange; rHIE: regional health information exchange system; CBPR: Community-based participatory research; eCRF: electronic case report forms.

Multimedia Appendix 2 Characterization of participating focus regions and their representatives during the development of the regional health information exchange system. The table represents the results from the questionnaire as well as the findings from the participant observations and informal interviews.

Multimedia Appendix 3 Chronology of the community-based participatory research (CBPR) workshops in each focus region and the achievements that were made during the workshops. rHIE: regional health information exchange system.

Multimedia Appendix 4 Community-based participatory research aspects of the collaboration between the participating health care facilities and the researchers according to the observational data in the 3 different focus regions. Source: project diaries.

Multimedia Appendix 5 Screenshots of the regional health information exchange system: (A) an example of a geriatric assessment electronic case report form, tandem stand, and (B) the comment function that supports free communication between the authorized health care providers.

Multimedia Appendix 6 Barriers to the use of the regional health information exchange system from the perspective of the users according to the results of the content analyses of the project diaries.

Multimedia Appendix 7 Facilitators of the use of the regional health information exchange system from the perspective of the users according to the results of the content analyses of the project diaries.

Multimedia Appendix 8 The participants’ agreement with certain statements categorized by various aspects of the technology acceptance model 2. To express their agreement, respondents could use a 5-point-Likert scale (n=12). HIT: Health IT.

## Abbreviations

CBPR: community-based participatory research
eCRF: electronic case report form
EHR: electronic health record
EMR: electronic medical record
GP: general practitioner
HIE: health information exchange
HIT: health information technology
ICT: information and communication technology
SRQR: Standards for Reporting Qualitative Research
TAM: technology acceptance model
