# Supplementary figures and images for: Evaluation of a Health Information Exchange System for Geriatric Health Care in Rural Areas: Development and Technical Acceptance Study
Source: JMIR Hum Factors. 2022 Sep 15;9(3):e34568. doi: 10.2196/34568 (PMC9523522; doi:10.2196/34568)

Multimedia  
Appendix 1

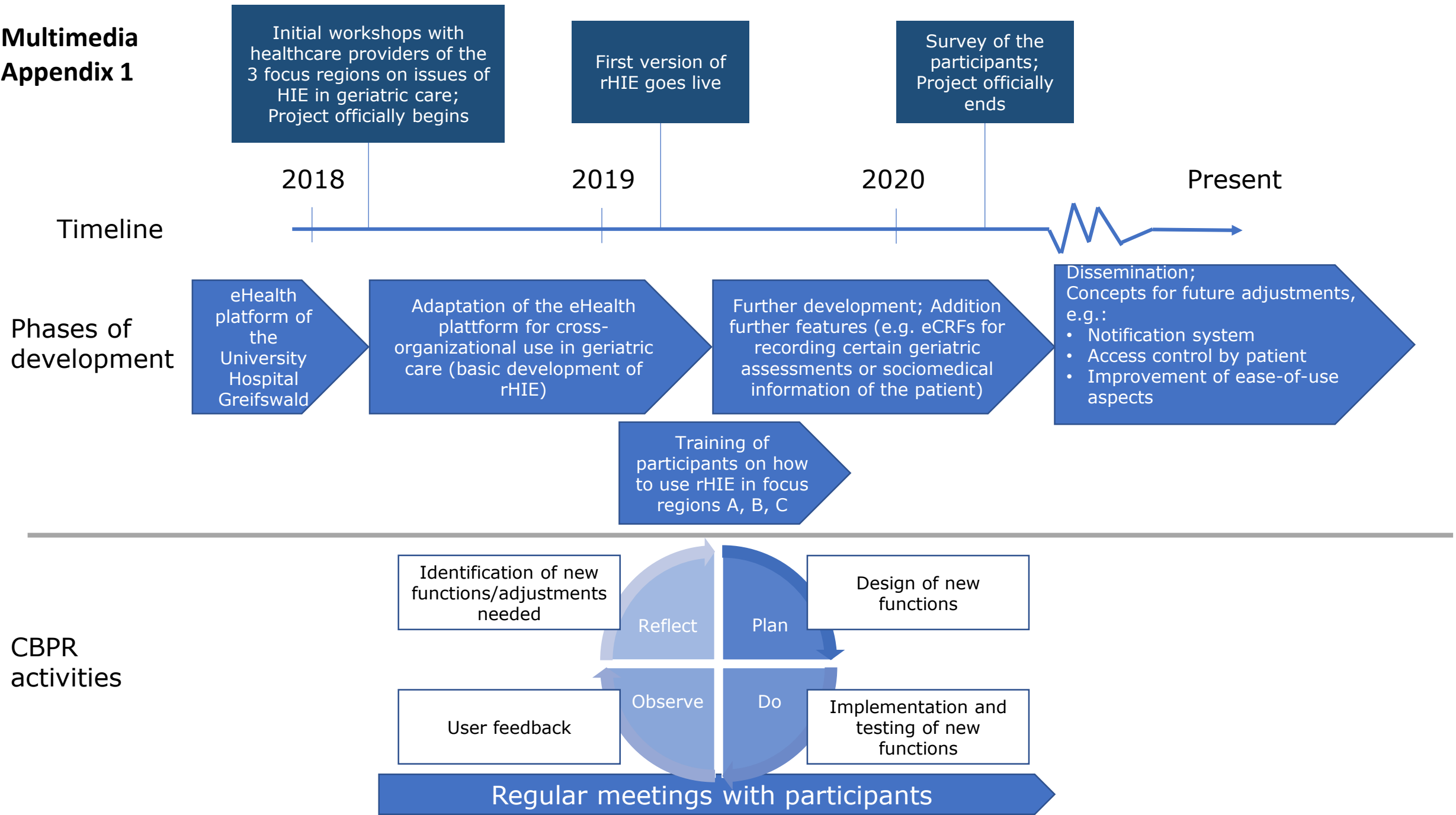

Supplement: Multimedia Appendix 1 [file humanfactors_v9i3e34568_app1.pdf]

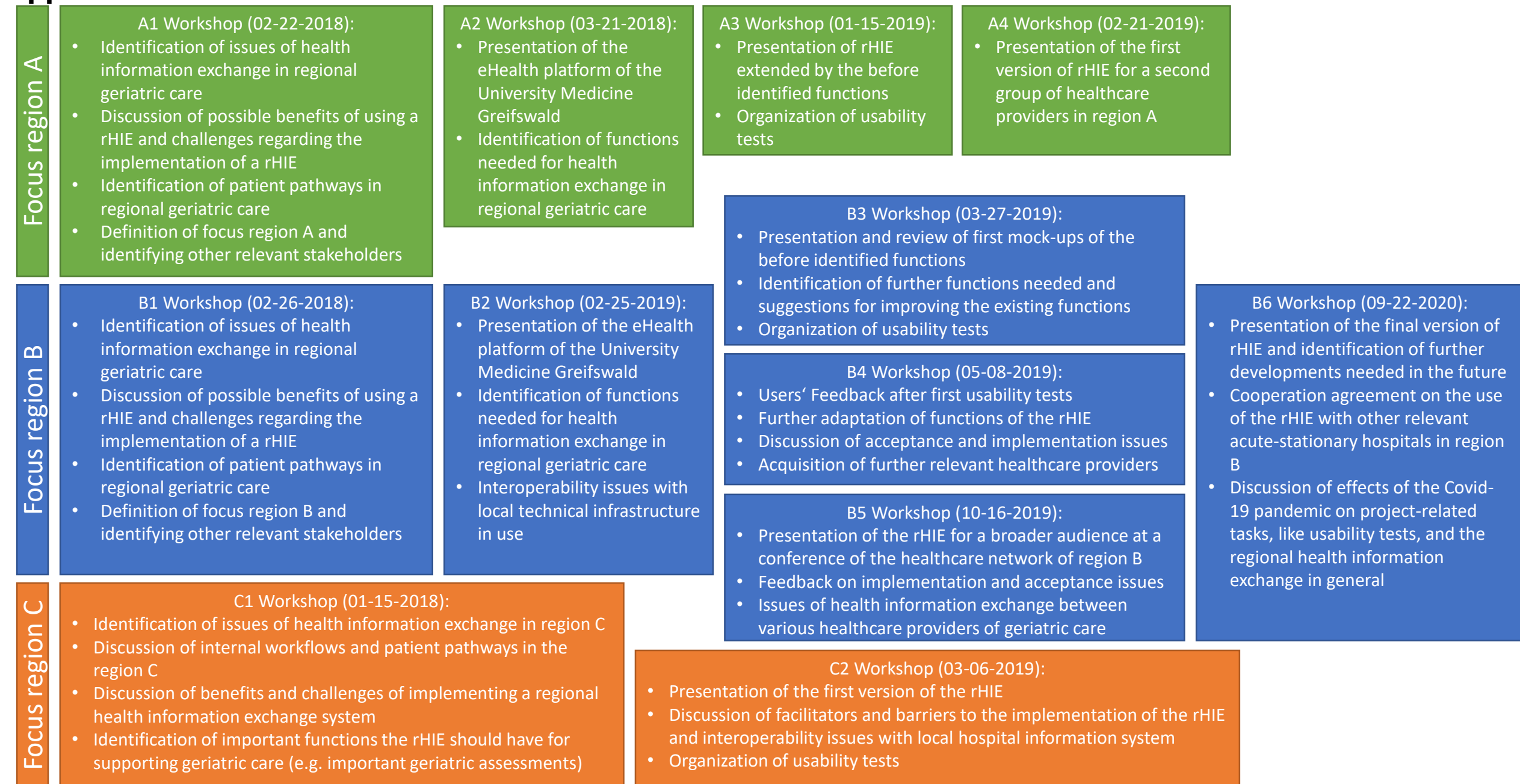

Supplement: Multimedia Appendix 3 [file humanfactors_v9i3e34568_app3.pdf]

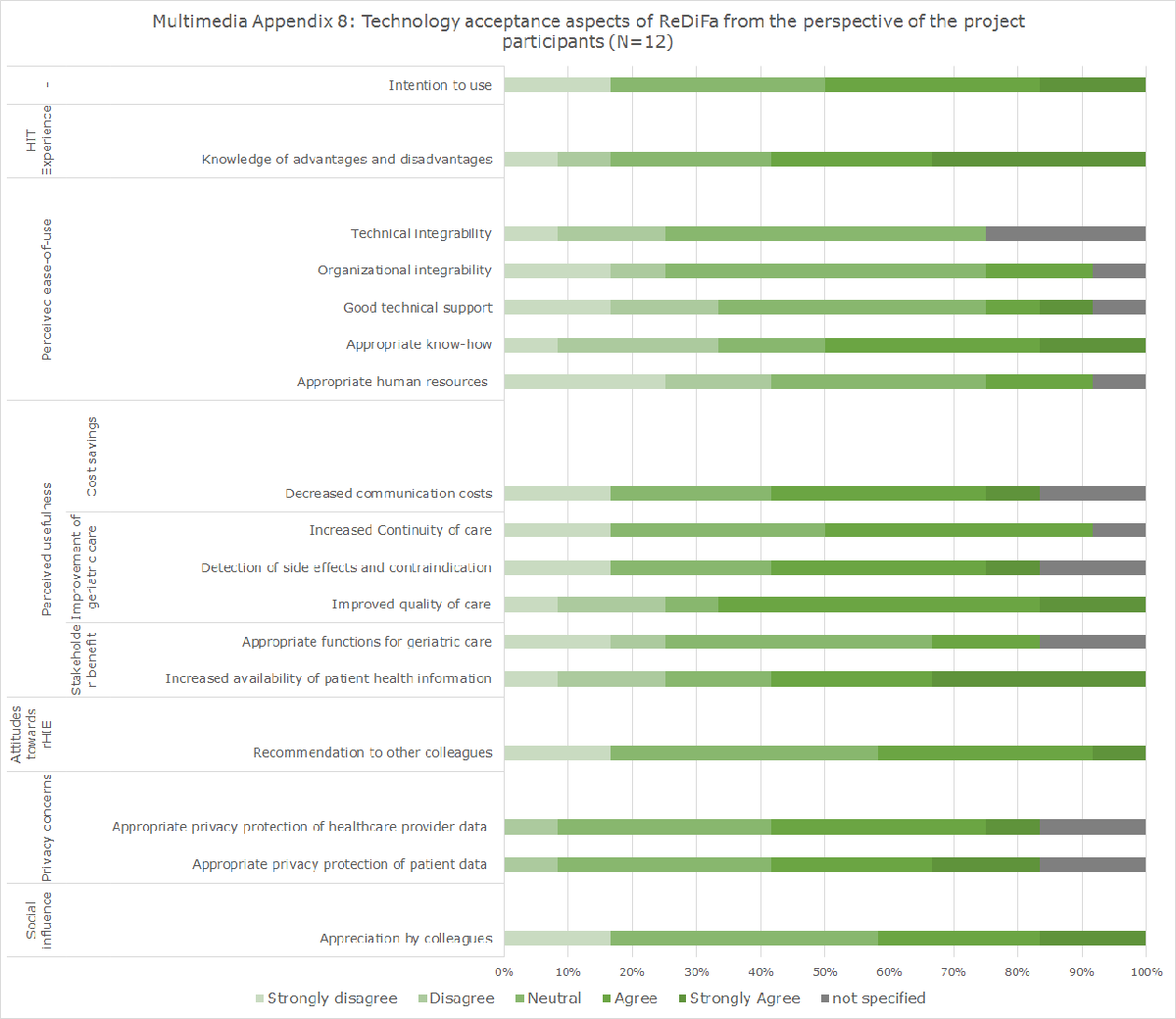

Supplement: Multimedia Appendix 8 [file humanfactors_v9i3e34568_app8.png]
