# Supplementary material for: Evaluation of a Health Information Exchange System for Geriatric Health Care in Rural Areas: Development and Technical Acceptance Study
Source: JMIR Hum Factors. 2022 Sep 15;9(3):e34568. doi: 10.2196/34568 (PMC9523522; doi:10.2196/34568)
Supplement: Multimedia Appendix 2 [file humanfactors_v9i3e34568_app2.pdf]

Multimedia Appendix 2

	Region A	Region B	Region C
**Participating healthcare facilities**			
	hospital without geriatric department	geriatric rehabilitation clinic	hospital with geriatric department
	GP practices	GP practice	geriatric day clinic
	occupational therapy practice	medical supply store	
	medical supply store	coordination office of the network	
	outpatient nursing service		

**Professions of the participating healthcare providers**			
	GP	GP	internist with geriatrics training working at a rehabilitation clinic
	occupational therapist	neurologist with geriatrics training working at a hospital	neurologist with geriatrics training working at a rehabilitation clinic
	nephrologist at a hospital	neuropsychologist with geriatrics training working at a hospital	nurse
	head of a hospital	therapy team (physio- and occupational therapy)	therapy team (physio- and occupational therapy)
	nurse	network coordinator	head of a day clinic
	employee of a medical store	employee of a medical store	
	internist with geriatrics training working at a hospital	care manager	

**Formal Healthcare Network**			
	yes	yes	no
**Geriatric focus of the network**			
	no	yes	n.a. (no formal network)
