# Supplementary material for: Evaluation of a Health Information Exchange System for Geriatric Health Care in Rural Areas: Development and Technical Acceptance Study
Source: JMIR Hum Factors. 2022 Sep 15;9(3):e34568. doi: 10.2196/34568 (PMC9523522; doi:10.2196/34568)
Supplement: Multimedia Appendix 4 [file humanfactors_v9i3e34568_app4.pdf]

Multimedia Appendix 4

CBPR aspects of the project-collaboration	Region A	Region B	Region C
**Relationship to the research team**			
	researchers are seen as a software provider, not as equal partners in a joint EHR development project	trusting, participative cooperation	trusting, participative cooperation
**Network identity**			
	hierarchical, focused on the primary care physician	participative, inter-professional	no formal network
**Conflict management**			
	non-transparent, distrustful	transparent, solution-orientated	non-transparent, solution-orientated
**Balance between practice and knowledge generation**			

	unbalanced	balanced	balanced
**Communication**			
	sporadic	steady, regular joint events	sporadic
**Continuity of the working process**			
	discontinuous	continuous	discontinuous
**Strategic planning**			
	no joint strategic planning	joint strategic planning	partial joint strategic planning
**Local leadership**			
	a hospital without a geriatric department and a GP without geriatric specialization.	a geriatric rehabilitation clinic and a GP specialized in geriatrics	a hospital with a geriatric department and a geriatric day clinic
**CBPR-Readiness**			
	initially interested, cancelled cooperation later	not interested initially but interested now	interested
