# Supplementary material for: Evaluation of a Health Information Exchange System for Geriatric Health Care in Rural Areas: Development and Technical Acceptance Study
Source: JMIR Hum Factors. 2022 Sep 15;9(3):e34568. doi: 10.2196/34568 (PMC9523522; doi:10.2196/34568)
Supplement: Multimedia Appendix 5 [file humanfactors_v9i3e34568_app5.pdf]

Multimedia Appendix 5

a)

c37.eHealth-Portal-UMG

Regionale Digitale Fallakte Wolgast

Online: Casemanager
Nils Pfeuffer

Basisdaten **Assessm. Selbstversorgung/Mobilität** **Assessm. Instrumentelle Aktivitäten** **Assessm. Kognition/Emotion** **Medizinische Übersicht** **Dokumente**

Formular anlegen:

Tandemstand/-gang

<< >>

Neumann, Michael
(männlich) *15.11.1938
Wolgaster Str. 50
17489 Greifswald
03834555687 (Telefon)

Tandemstand/-gang

Nähere Informationen zum Tandemgang und -stand finden Sie auf den Seiten des Kompetenzzentrums für Geriatrie.

Folgende Standpositionen können unterschieden werden: a) Hüftbreiter Stand, b) geschlossener Stand, c) Semitandemstand, **d) Tandemstand**, e) Einbeinstand.

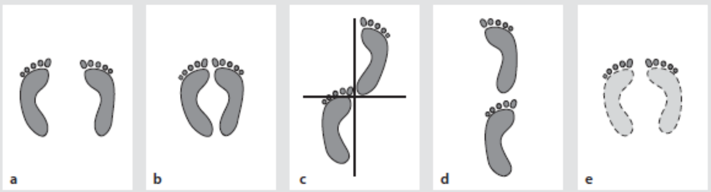

(Richter, K. et al., Der ältere Mensch in der Physiotherapie, 2016)

Kommentare zu diesem Fall:

Ihr Kommentar

Kommentar speichern

Tandemstand

Hinweis: Patient soll 10 Sekunden im Tandemstand stehen, d.h. beide Füße in einer Linie hintereinander, wobei die Ferse des einen Fußes die Spitze des anderen berührt. Arme hängen locker, Augen geöffnet. Hilfe bei Einnahme der Position erlaubt. Jedes Umsetzen der Beine beendet den Versuch. Die beste Umsetzung von 3 Versuchen wird gewertet.

Tandemstand 1. Versuch

Tandemstand 2. Versuch

Tandemstand 3. Versuch

Tandemgang

Hinweis: Patient soll 8 Schritte im Tandemgang setzen, d.h. auf einer (am besten markierten) Linie je einen Fuß so vor den anderen setzen, dass die Ferse des einen Fußes die Spitze des anderen berührt. Jedes Umsetzen der Beine, ein Abstand der Ferse vonmehr als 2 cm von der Fußspitze, oder eine seitliche Abweichung von mehr als einer halben Fußbreite aus der Spur beendet den Versuch. Gezählt wird die maximale Anzahl korrekt gesetzter Schritte in 3 Versuchen.

Tandemgang 1. Versuch

Tandemgang 2. Versuch

Tandemgang 3. Versuch

in Arbeit | i | Speichern | Zurück

Neumann, Michael, Geb.: 15.11.1938 (81)

Version: 2018-8 © 2007-2019 celsius37.com AG

b)

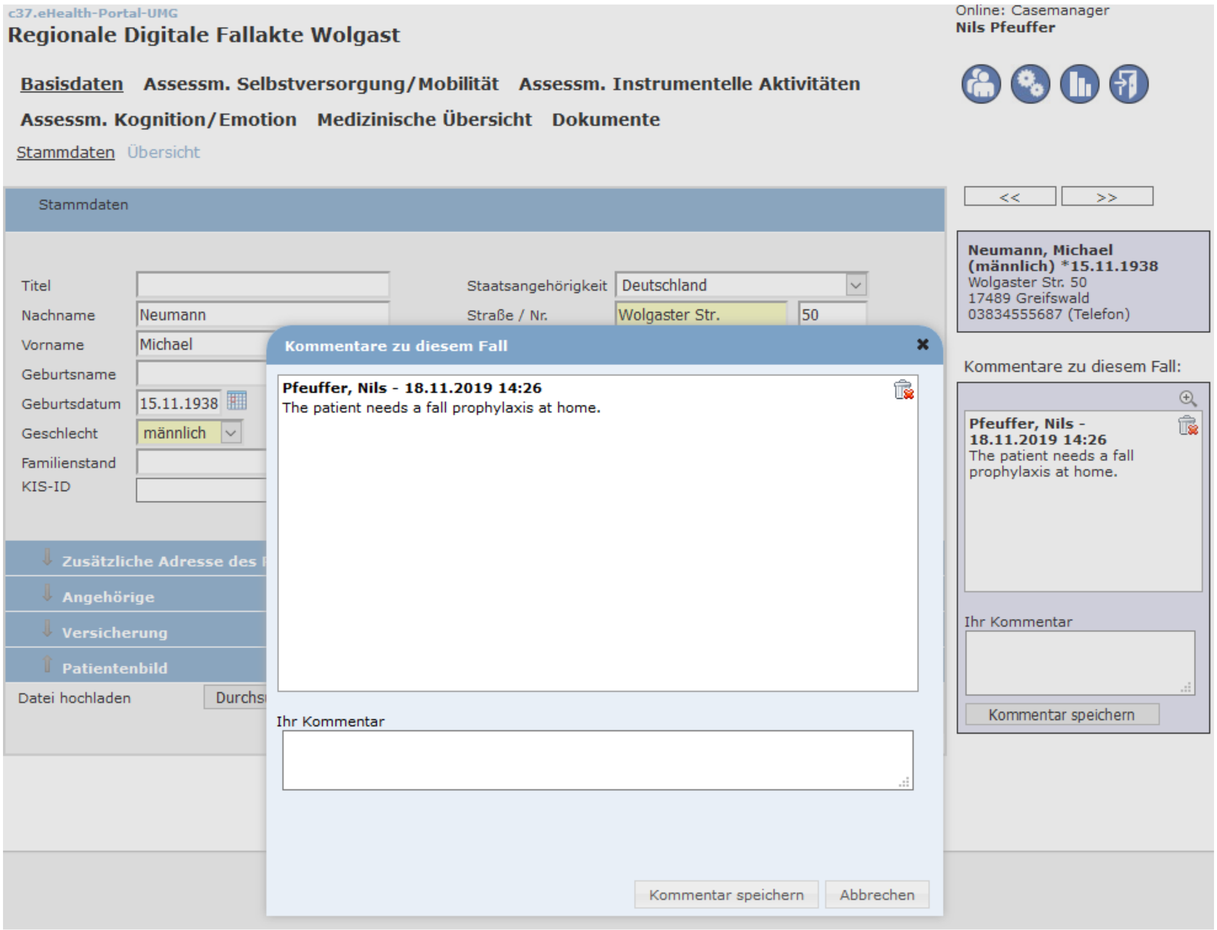
c37.eHealth-Portal-UMG
Regionale Digitale Fallakte Wolgast
Online: Casemanager
Nils Pfeuffer
Basisdaten
Assessm. Selbstversorgung/Mobilität
Assessm. Instrumentelle Aktivitäten
Assessm. Kognition/Emotion
Medizinische Übersicht
Dokumente
Stammdaten
Übersicht
Stammdaten
Titel
Nachname
Neumann
Vorname
Michael
Geburtsname
Geburtsdatum
15.11.1938
Geschlecht
männlich
Familienstand
KIS-ID
Staatsangehörigkeit
Deutschland
Straße / Nr.
Wolgaster Str.
50
Angehörige
Versicherung
Patientenbild
Datei hochladen
Kommentare zu diesem Fall
Pfeuffer, Nils - 18.11.2019 14:26
The patient needs a fall prophylaxis at home.
Ihr Kommentar
Kommentar speichern
Abbrechen
Neumann, Michael
(männlich) *15.11.1938
Wolgaster Str. 50
17489 Greifswald
03834555687 (Telefon)
Kommentare zu diesem Fall:
Pfeuffer, Nils -
18.11.2019 14:26
The patient needs a fall
prophylaxis at home.
Ihr Kommentar
Kommentar speichern
