# Supplementary material for: Evaluation of a Health Information Exchange System for Geriatric Health Care in Rural Areas: Development and Technical Acceptance Study
Source: JMIR Hum Factors. 2022 Sep 15;9(3):e34568. doi: 10.2196/34568 (PMC9523522; doi:10.2196/34568)
Supplement: Multimedia Appendix 6 [file humanfactors_v9i3e34568_app6.pdf]

**Multimedia Appendix 6**

| No. | Barrier | Description | Examples of project diary entries |
|---|---|---|---|
| | | | |
| **1** | **Availability of information** | | |
| | | There is no automatic synchronization of patient data between the local systems and rHIE yet. So, the timely availability of information still depends on when individual users transfer the data. | "the recording on the rHIE should be done immediately [after a patient encounter] so that no unrecognized cases accumulate"<br>"The diagnoses must be constantly updated manually by several practitioners, which is on the one hand expensive and on the other hand a source of mistakes, because it is doubtful that the patient information on rHIE is up-to-date."<br>"The medical transcriptionist receives the [paper-based] patient record after the patient has been discharged and only then enters the data into rHIE."<br>"Double data entry into two parallel systems is considered the greatest barrier." |

| No. | Barrier | Description | Examples of project diary entries |
|---|---|---|---|
| | | | |
| **2** | **Technical issues** | | |
| | | Required functions or input fields are missing;<br>Technical errors during using or testing;<br>Complicated user registration process;<br>Loss of login data;<br>Homepage was temporarily unavailable;<br>Disconnections during mobile use<br>Problems with the web browser or web browser settings | "[Occupational therapist] is asking for another master data field, in addition to 'contact of a relative', to also record caregivers of the patient, [because] relatives and caregivers are often different persons"<br>"There is no way to identify other therapists or even open a direct communication channel."<br>"GP was requested to confirm the user certificate during his login on rHIE several times. […]GP is the first one to use 'Safari' as a web browser."<br>"Occupational therapist did not install his client certificate, so rHIE was not available" |
| **3** | **Lack of standardization** | | |
| | | Differences in documenting patient´s information, e.g. different types of medication record between inpatient and outpatient providers. | "The rehabilitation clinic does not use the 'nationwide medication plan', but the general practitioners do. As a result, prescribed medications are documented to a different level of detail." |

| No. | Barrier | Description | Examples of project diary entries |
|---|---|---|---|
| | | | |
| **4** | **Lack of communication and coordination** | | |
| | | rHIE should provide automatic notifications to inform other users about updates or uploads of treatment data or documents. | "There is no way to determine further therapists or even open a direct communication channel."<br>"The problem of how to communicate with other relevant healthcare providers who have no access to rHIE was discussed." |
| **5** | **No area-wide use of rHIE** | | |
| | | Often not all relevant healthcare professionals of one patient have access to rHIE. So, information gaps are still possible. | "It is a waste of time to record patient health information on rHIE if no one is looking up this information."<br>"The contact with the geriatric clinic, which is formally integrated in the medical network, has not yet been established. Therefore, they do not yet have access to rHIE." |

| No. | Barrier | Description | Examples of project diary entries |
|---|---|---|---|
| | | | |
| **6** | **Ease-of-use issues** | | |
| | | Different aspects of ease-of-use:<br>Mobile use of rHIE is dependent on local internet connectivity;<br>Some mandatory fields are not necessary;<br>Missing input fields for entering contact details of caregivers;<br>The medical data overview could be more informative;<br>No preview for uploaded documents | "Pop-up windows which ask 'Do you want to continue without saving' are missing."<br>"The application could be easier to use (e.g. drag & drop for uploading files faster)."<br>"Medical information entries appear in the summary only with their category title (e.g. active main diagnosis), but without a specific descriptor (e.g. ICD-10 code or title)."<br>"No image preview is available for uploaded documents." |

| No. | Barrier | Description | Examples of project diary entries |
|---|---|---|---|
| | | | |
| **7** | **Costs for using rHIE** | | |
| | | Data entry is said to take too long, especially for data which has to be entered twice: in the local EMR and the rHIE. | "For test purpose, the GP and his practise team gathered patient health information and entered the data on rHIE outside of working hours]. They complained that, during working hours, this procedure would always block a workstation. The GP estimates an average time of 45 minutes to create a new electronic patient record on rHIE and transfer all the required treatment information into rHIE." |

| No. | Barrier | Description | Examples of project diary entries |
|---|---|---|---|
| | | | |
| **8** | **Availability of resources** | | |
| | | There are differences between various facilities in terms of the availability of resources, e.g., human resources. | "The network coordination office reports that two medical typists will be hired for using rHIE from May, and later from June."<br>"Scarce human resources - CD recommends refinancing doctoral students or similar from the expense allowance, who then could operate the rHIE."<br>"For test purposes, the GP and his practise team gathered patient health information and entered the data on rHIE outside of working hours]. They complained that, during working hours, this procedure would always block a workstation. The GP estimates an average time of 45 minutes to create a new electronic patient record on rHIE and transfer all the required treatment information into rHIE." |
| **9** | **Lack of trust** | | |
| | | The willingness to participate in the rHIE-project seems to depend on the level of trust within the individual networks. | "One participant from Region A […] did not trust the other members of the network."<br>"In Region A, the research team had to discuss the development of rHIE in two separate working groups: one working group consisted only of physicians and the other consisted of therapists and other healthcare professionals." |

| No. | Barrier | Description | Examples of project diary entries |
|---|---|---|---|
| | | | |
| **10** | **Lack of cooperation** | | |
| | | Some participants do not take advantage of the researchers' support. | "I again offered to support the GP in entering patient health information on site, but the offer was not used." |
| **11** | **Privacy concerns** | | |
| | | Industrial espionage;<br>Uncertainty about the legal regulation of data protection in the field of eHealth;<br>Missing implementation of a rights and role-based access logic | "Mostly in Region A the participants were concerned about the privacy of the users. They fear that competitors could use the rHIE for a kind of industrial espionage."<br>"The GP and the network coordinator agreed that a rights and roles concept should be implemented in rHIE so that certain user groups can only view and edit certain types of documents that they have access to."<br>"It is unclear whether the legal framework exists to later be able to use rHIE in practice." |

| No. | Barrier | Description | Examples of project diary entries |
|---|---|---|---|
| | | | |
| **12** | **Organization within the network** | | |
| | | Differences in the organization of the networks, e.g. hierarchical vs. an inter-professional structure. | "In Region A, the medical board members organized and performed the testing of rHIE on behalf of the other participating network members."<br>"Within the network of Region B there is an interdisciplinary task force that deals solely with issues of cross-organizational cooperation [‘task force Discharge Management’]." |
| **13** | **Low computer literacy** | | |
| | | Differences in the individual readiness to use HITs can influence the acceptance of the individual providers. | "[GP complaints:] Documents would first have to be exported from the PMS or HIS, which is more complicated than expected, and then uploaded again on rHIE." |
