# Supplementary material for: Evaluation of a Health Information Exchange System for Geriatric Health Care in Rural Areas: Development and Technical Acceptance Study
Source: JMIR Hum Factors. 2022 Sep 15;9(3):e34568. doi: 10.2196/34568 (PMC9523522; doi:10.2196/34568)
Supplement: Multimedia Appendix 7 [file humanfactors_v9i3e34568_app7.pdf]

Multimedia Appendix 7

No.	Facilitator	Description	Examples of project diary entries
**1**	**Adaptability to local needs**		
		Modular structure of the rHIE enables adaptation to local needs; Web-based structure enables access to various facilities regardless of the technical equipment (only Internet access is required); rHIE can be used by all patients regardless of their health insurance company; rHIE provides better data security than other alternatives (e.g. paper-based dockets, USB flash drives, WhatsApp, etc.) and	"Modular structure allows network-specific tailoring [of the rHIE] to support data exchange according to local standards or processes."<br>"Within the network of Region B there is an interdisciplinary task force that deals solely with issues of cross-organizational cooperation ['task force Discharge Management']."<br>"[The rHIE] in its present form is already an improvement to what the taskforce 'Discharge Management' has so far been conceived for the problem of data exchange in the patient transfer process [cross-institutional paper-pencil-based docket, that has not been finalized yet]."<br>"Therapy team raises the question of whether it might be technically easier to exchange patient health information via USB flash drives."<br>"However, the chief doctor sees the benefits of rHIE in comparison with the EHR [of individual insurance companies],

No.	Facilitator	Description	Examples of project diary entries
		also improves the availability of information.	because the rHIE can be used for all patients and can be specifically designed for the local settings.”
No.	Facilitator	Description	Examples of project diary entries
**2**	**High computer literacy**		
		Differences in the individual readiness to use HITs can influence the acceptance of the individual providers.	“GP seems to be very IT-savvy, because GP emphasized the importance of digitizing all documents of his practice.”<br>“Overall, GP finds the use of the rHIE is not complicated and […] he also considers the effort [required to use] the rHIE to not be demanding.”<br>“[GP complaints:] Documents would first have to be exported from the PMS or HIS, which is more complicated than expected, and then uploaded again on rHIE.”

No.	Facilitator	Description	Examples of project diary entries
**3**	**Understanding rHIE as a health information exchange system**		
		Users who see rHIE more as a health information exchange system rather than a comprehensive EHR, are less concerned about the effort required to use rHIE.	“GP finds it clearer [to use rHIE as a health information system] than using rHIE as a second record alongside his local patient record”<br>“At this point concerns came up that this is all too much. Chief doctor does not want to have ‘nursing stories’ on rHIE.”<br>“With the help of photographs that showed a centimetre-thick paper record, a Chief doctor expressed that transferring all treatment information into rHIE is too time-consuming.”
**4**	**Trust among network partners**		

		The willingness to participate in the rHIE-project seems to depend on the level of trust within the individual networks.	In Region B: "Good working relationship, communication and trust, interdisciplinary team discussions (including occupational therapy and nursing)."
No.	Facilitator	Description	Examples of project diary entries
**5**	**Availability of resources**		
		There are differences between various facilities in terms of the availability of resources, e.g., human resources.	"Overall, GP finds the use of the rHIE is not complicated and […] he also considers the effort [required to use] the rHIE to not be demanding."
